# Is Bodily Experience an Epiphenomenon of Multisensory Integration and Cognition?

**DOI:** 10.3389/fnhum.2019.00316

**Published:** 2019-09-11

**Authors:** Josselin Baumard, François Osiurak

**Affiliations:** ^1^Normandie Univ, UNIROUEN, CRFDP (EA7475), Rouen, France; ^2^Laboratory for the Study of Cognitive Mechanisms (EA 3082), University of Lyon, Lyon, France; ^3^French University Institute, Paris, France

**Keywords:** action, bodily awareness, body schema, body image, epistemology, multisensory integration, sense of ownership, sense of agency

## Introduction

Having a physical body is not sufficient to experience the feeling of having a body. This somewhat staggering assumption has long been demonstrated by studies on phantom limbs (Ramachandran, 1998), distortions of body image following right brain damage (Hécaen and Ajuriaguerra, 1952), and experimentally induced body illusions (Botvinick and Cohen, 1998; Petkova and Ehrsson, 2008). A critical issue is now to understand which mechanisms underlie bodily experience (de Vignemont, 2010), a prerequisite to develop studies on tool incorporation, and neurorehabilitation. That being said, the orientation of research strongly depends on the selected epistemological options. The present work aims at discussing two epistemological options, one being representational (i.e., bodily experience relies on the activation of specific cognitive modules devoted to body representations), and the other being structuralist (i.e., bodily experience is an epiphenomenon of both multisensory integration and cognition).

## Defining Body Schema and Body Image

Classical taxonomies (Sirigu et al., 1991; Schwoebel and Coslett, 2005) have made a distinction between three body representations. First, the body schema is an immediate sensorimotor representation that specifies the relative positions of body parts in space over time (Buxbaum, 2001). Second, body semantics are of conceptual and linguistic nature, and describe the functions and categories of body parts (e.g., both the wrist and elbow are joints). Third, the body structural description is mainly of visual nature and provides individuals with knowledge on the normal structure of the body (e.g., relative positions of body parts; Goldenberg, 1995). It is a long-term body representation that may also be broken down into a general body image (e.g., knowing that all humans have two arms) and an individual body image (i.e., the stable representation of one's own body over time). The latter implies that individual experience plays a key role in body representation (i.e., the habitual body; Merleau-Ponty, 1945). Due to the conceptual ambiguity of these concepts (de Vignemont, 2010), we shall use the “bodily experience” label as a whole category encompassing body schema and body image and, more generally, the experience of having a body.

## Epistemological Issues

The abovementioned taxonomy admits that distinct cognitive modules are devoted to specific body representations. Nevertheless, “*there are so many bodily disorders, and therefore so many possible dissociations, that one would end up with an almost infinite list of body representations”* (de Vignemont, 2010, p. 7). In this view, the virtually infinite multiplication of cognitive modules would result in unfalsifiable theories of bodily experience (see also De Vignemont, 2007). It follows that a scientific theory should rely on a limited number of cognitive modules. However, if that is so, why are there so many different bodily disorders (de Vignemont, 2010)? Perhaps one solution would be to consider theoretical options of structuralism, an epistemological account initially developed in linguistics (De Saussure, 1915), and anthropology (Lévi-Strauss, 1958), and occasionally applied to neurological patients (Sabouraud, 1995).

**Figure 1:**
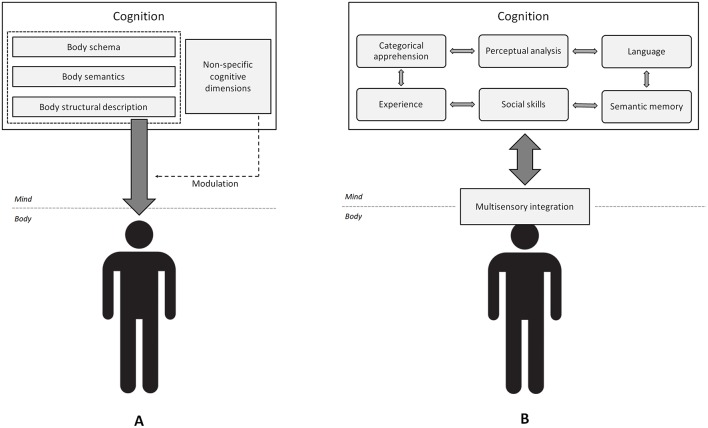
Two epistemological accounts of bodily experience. **(A)** The cognitive account of bodily experience. Specific body representations determine bodily experience, while additional, non-specific cognitive components modulate the expression of these representations. **(B)** The structuralist account of bodily experience. There are no body-specific representations. Instead, all of the cognitive processes (not necessarily body-specific) interfere with multisensory integration, which results in bodily experience. Cognitive dimensions also interact with each other (small gray bidirectional arrows), resulting in various phenomena. Adopted from https://pixabay.com/fr/service/license/.

The main assumptions of structuralism are as follows. (1) The human mind consists of a minimum set of modular components (i.e., the structure), the number of which is limited by their universality (e.g., all humans are capable of language independently of the multiplicity of languages). This is consistent with the modularity and universality of mind assumed in cognitive psychology (Marr, 1982; Fodor, 1983). However, (2) the diversity of individual experiences does not reflect the activity of specific cognitive modules but rather is incidental and conjuncture-dependent: Different individual experiences may lead to infinite variations of individual psychological conformations (e.g., the painting), and yet the underlying structure should be the same across individuals (e.g., the canvas). (3) Components of the mind are interdependent rather than independent, sequential and hierarchical. “Heterarchy” may better reflect the complexity and non-linearity of brain activity (Fuster, 2009). (4) By contrast with strict modularity, sensations are processed by all of the components simultaneously (e.g., one familiar tool is simultaneously the object of both semantic and technical reasoning; Osiurak, 2014). This amounts to considering that components of the mind *interfere* with one another (i.e., the interference hypothesis) in the construction of phenomena (e.g., human written language may result from the interaction of language and technics; Gagnepain, 1990). On this account, perhaps bodily experience does not reflect specific body representations, but rather results from the interaction of all human cognitive skills (Figure 1).

## Implications for Theories of Bodily Experience

Even though structuralism is questionable for being too holistic, over the past few years embodied cognition experiments provided data consistent with the interference hypothesis. But before demonstrating relationships between bodily experience and any cognitive mechanism, it is necessary to delineate specific neurological mechanisms underlying bodily experience. In this regard, it is argued that multisensory integration is a prerequisite to experiment the unity and continuity of the body and self, and that interference with additional cognitive dimensions may underlie various body-related phenomena.

### Multi-Sensory Integration as a Body-Specific Process?

Before the advent of cognitive architectures, bodily experience has long been viewed by neurologists as an epiphenomenon of multisensory integration (i.e., the combination of sensations arising from different modalities and brain regions; Bonnier, 1905; Head and Holmes, 1911; see also de Vignemont, 2010; Longo and Haggard, 2012a,b). The latter is at the root of body unity (e.g., one can perceive her/his hand as a unitary body part because she/he sees and feels it at the same point in space) and makes it possible to distinguish between self and non-self (e.g., objects, others) stimulations. More recent body illusion experiments have revived and extended this multisensory account of body ownership (Botvinick and Cohen, 1998; Petkova and Ehrsson, 2008).

Multisensory integration depends on the activity of multiple brain regions including the sensory, premotor, posterior parietal, temporal superior, and internal cortex (Wiener, 1996; Calvert and Thesen, 2004; Petkova et al., 2011a; Ursino et al., 2014; Yau et al., 2015). Remarkably, these regions are not specific to bodily experience for they are also involved in cognitive functions like memory, visuospatial, praxis, and social skills. It is then plausible that these brain regions are actually not body-specific but contribute to bodily experience.

### Cognition and the Body

This section enumerates, non-exhaustively, findings/hypotheses that are in line with the interference hypothesis (i.e., the interaction between cognition and bodily experience).

#### Bodily Experience and Perceptual Analysis

It has long been assumed that body schema/image is independent from perceptual analysis. Nevertheless, well-known works have demonstrated that the physical characteristics of the body have an impact on visuospatial analysis (Proffitt, 2006). Likewise, motor imagery (i.e., the ability to mentally simulate movements of specific body parts) presumably involves the body schema but is sensitive to peripheral bodily conditions like chronic pain (Breckenridge et al., 2019). Therefore, body representations seem to be highly dependent on immediate bodily experience, and it is no longer possible to admit full independence between personal, and extrapersonal perception.

#### Bodily Experience and Action

In the field of apraxia, categorical apprehension (i.e., the ability to select and combine parts of multipart objects into a whole configuration) deficits may account for both apraxia of tool use and visuo-imitative apraxia (Goldenberg, 2009), two disorders that have been explained by either body schema (Buxbaum, 2001), or body image deficits (Goldenberg, 1995). Remarkably, categorical apprehension may be the direct psychological expression of the particular neuronal architecture of the left parietal lobe (rather than a specific cognitive module), which is why it may apply to both body, and non-body stimuli indiscriminately (i.e., body parts and objects). In the same vein, technical reasoning (i.e., the ability to infer tool/object characteristics that are relevant to achieve a given goal; Osiurak, 2014) might condition the selection of body parts during action (e.g., one may use her/his nails to play scratch-card games because nails have the same properties as coins to achieve the goal of scratching).

#### Bodily Experience and Language

Interestingly, a similar differentiation/combination function prevails in the field of linguistics (De Saussure, 1915), and presumably underlies categorization and concept formation (i.e., the ability to identify and group recurrent information across infinite experiences; Vallila-Rohter and Kiran, 2015). It is not surprising then, that correlations are frequently observed between gestures conveying meaning on the one hand, and language on the other hand (Vingerhoets et al., 2013). Actually, many works consistently demonstrated the bidirectional relationships between language and the body, especially action (Schwartz et al., 2008; Shebani and Pulvermüller, 2018), and body part localization (Mattioni and Longo, 2014). Perhaps bodily experience not only develops under the influence of language, but also varies greatly in everyday life by the mere fact of thinking and talking.

#### Bodily Experience and Semantic Memory

Broadly speaking, body semantics correspond to knowledge about the body and are independent from the body schema. Nevertheless, these representations are more intermingled than expected. Conceptual knowledge on body parts grows as a function of their involvement in action (Auclair Jambaqué and Jambaqué, 2015), and children with spinal cord injury may show selective deficits of body image (Salvato et al., 2017). It follows that body semantics are not the mere result of explicit, didactic learning but also of embodied, individual experience. The fact that body image can be selectively impaired in adults can be understood as an effect of culture-dependent brain plasticity. After all, partially different brain regions may underlie English and Greek in bilingual individuals and yet, they are not the expression of completely different cognitive processes (Ekiert, 2003). This embodied account of body image predicts that action-based tasks should be as efficient as semantic-based tasks in the rehabilitation of body image deficits.

#### Bodily Experience and Social Skills

Another property of semantic memory—and hence body semantics—is that it is a shared, collective memory acquired through social interactions. The fact that similar brain regions represent the self and others (Kruse et al., 2016) supports the hypothesis of a socially grounded bodily experience. Besides, the observation of others shapes the multisensory peripersonal space (Pellencin et al., 2018). Likewise, the estimated metrics of someone else's body depends on social features like gender (Linkenauger et al., 2017). In this regard, it is likely that attitudes toward social partners influence bodily experience, especially since the emotional valence of stimuli has an influence on movement control (so, perhaps, on body schema; Esteves et al., 2016). This might imply that the quality of the relationship between a patient and its physical therapist have a direct effect on rehabilitation, in that positive attitudes toward the therapist may reconfigure peripersonal space in itself.

#### Bodily Experience and Individual Experience

Studies on body-swapping illusions (Petkova and Ehrsson, 2008) have demonstrated that they are limited (de Vignemont and Farnè, 2010): The illusion does not work with objects that are not body-shaped, and the feeling of owning the new body occurs only from a 1st person perspective (Petkova et al., 2011b). Nevertheless, with regard to the plasticity of perception (Sachse et al., 2017), it is probably because we are used to experiment our body in a 1st person perspective (i.e., the habitual body). Indeed, body-related tasks are influenced by both individual habits (Isaac and Marks, 1994), and the experience of either the first or the third perspective (Edwards et al., 2019). Contrary to long-standing beliefs, there may be no limit to the plasticity of bodily experience with the possible exception of experience (i.e., the habitual body). This “habitual body” might correspond to the concept of “body model” (i.e., the implicit representation of the usual size and shape of one's own body parts), and can be understood as the phenomenological expression of the somatotopic organization of the somatosensory cortex (Longo and Haggard, 2010). It should be acknowledged that the crucial role of individual experience in stabilizing the body model is not incompatible with the existence of a basic, innate organization of the brain acquired through phylogenesis (Longo et al., 2012).

## Potential Implications for Neurorehabilitation

As mentioned in the introduction, theoretical options should have implications for clinical practice, especially neurorehabilitation. The structuralist account of bodily experience posits that the latter is an epiphenomenon of both multisensory integration and cognitive processes that are not body-specific. On this ground, future research on clinical syndromes may include extensive testing of both multisensory and cognitive processing. Indeed, setting up therapies implies the upstream demonstration of the level of impairment. In the absence of a consensual, unified framework for the study of bodily experience (de Vignemont, 2010), this would involve thorough testing of cognition and body representation.

Furthermore, two strategies could be tested. The first strategy focusing on multisensory integration would aim at modifying bodily experience by modulating one or several afferent sensory inputs (e.g., enrichment or impoverishment). This corresponds to most of the strategies currently tested based on the now well-established role of multisensory integration in bodily experience (e.g., Chokron et al., 2007; Moseley et al., 2008; Diers et al., 2013). A second, complementary strategy based on the interference hypothesis could consist in testing the influence of non-specific cognitive processes on abnormal bodily experience. This could include, at least, perception (e.g., does modifying the environment of the body modulate bodily experience and improve symptoms?), action (e.g., does tool use action improve symptoms?), language (e.g., does talking and thinking modify bodily experience?), semantics (e.g., does mental imagery of the body improve symptoms in peripheral syndromes? Does the modulation of peripheral afferent information help asemantic patients drawing body parts?), social skills, and emotion (e.g., does the empathy or emotional state of the patient have an impact on symptoms?), and individual experience (e.g., does the intensity of symptoms vary as a function of previous individual habits?).

Furthermore, seeing the extensive list of body-related disorders (de Vignemont, 2010), it seems necessary to test which therapy is effective on which syndrome. For instance, it is unlikely that the same strategies may apply to both peripheral neurological conditions, and syndromes caused by brain lesions. In return, this could lead to categorize body-related disorders depending on which therapy is effective, and hence to better understand the either common or different underlying nature of seemingly different body-related disorders (e.g., if one and the same treatment is effective on both eating disorders and somatoparaphrenia, one might consider that these conditions share a common denominator). Ultimately, neurorehabilitation studies could lead to either confirm or invalidate the hypothesis that bodily experience is an epiphenomenon of multisensory integration and cognition.

## Conclusion

The now well-documented permeability of bodily experience and cognitive functioning raises a critical epistemological issue. On a cognitive account of bodily experience, one could argue that body representations exist, and may plastically change during action (Maravita et al., 2003). Nevertheless, if representations are plastic to that point, then one may also wonder what the nature of these representations is, and which specific function they subserve. An alternative structuralist account would be to consider body representations as the consequence rather than the cause of bodily experience and cognition. On this ground, it is proposed that bodily experience is an epiphenomenon of multisensory integration (likely the most body-specific process), and cognition (Figure 1). This is not fully in line with theories of embodied cognition because it amounts to considering that cognition shapes the body as much as the body shapes the mind, whereas embodied cognition accounts generally posit that the body shapes cognition. Demonstrating abnormal bodily experience in the context of completely normal sensory, and cognitive functioning would stand against the hypothesis defended here.

## Author Contributions

All authors listed have made a substantial, direct and intellectual contribution to the work, and approved it for publication.

### Conflict of Interest Statement

The authors declare that the research was conducted in the absence of any commercial or financial relationships that could be construed as a potential conflict of interest.
